# The Translational Dermatology Initiative: Aiming at a New Disease Classification of Inflammatory Skin Diseases

**DOI:** 10.1016/j.xjidi.2025.100381

**Published:** 2025-05-13

**Authors:** Pontus Jonsson, Anna Caroline Pilz, Heydar Maboudi, David Ranzinger, Paul Wagner, Larissa-Nele Schaffert-Stone, Caecilia Burg, Mahsa Shahidi Dadras, Maria Bradley, Franziska Schauer, Christoph Mathis Schempp, Natalie Garzorz-Stark, Stefanie Eyerich, Kilian Eyerich

**Affiliations:** 1 Unit of Dermatology, Department of Dermatology and Venereology, Karolinska University Hospital, Stockholm, Sweden; 2 Division of Dermatology and Venereology, Department of Medicine Solna, Karolinska Institutet, Stockholm, Sweden; 3 Center for Molecular Medicine, Karolinska Institutet, Stockholm, Sweden; 4 Department of Dermatology and Venerology, Medical Center and Faculty of Medicine, University of Freiburg, Freiburg, Germany; 5 Institute for Immunodeficiency, Center for Chronic Immunodeficiency, Medical Center, University of Freiburg, Freiburg, Germany

**Keywords:** Classification, Individualized medicine, Inflammatory skin disease, Precision medicine, Stratified medicine

## Abstract

Although precision medicine is at least partially realized in dermato-oncology, the field of dermatoimmunology comprising inflammatory skin diseases is only at the step from traditional toward stratified medicine. This lack of innovation leaves clinically relevant questions unanswered, including predicting the personal likelihood of therapeutic success as well as the risk of drug-related adverse events or the development of comorbidities. The translational dermatology initiative hypothesizes that these shortcomings are due to the subjective nature of the current disease ontology, which does not address the heterogeneity and dynamics of diseases. By integrating deep clinical phenotyping and repetitive multiomics analyses of tissue and circulation of patients covering the whole spectrum of chronic skin inflammation independent of the traditional disease nomenclature, the translational dermatology initiative creates a high-quality dataset optimized for machine learning. The aim of the translational dermatology initiative is to reclassify inflammatory skin diseases on the basis of objective molecular events that enable prediction of clinically meaningful outcome variables. The translational dermatology initiative is currently recruiting at 2 centers (Freiburg and Stockholm), with the aim to expand this into a global initiative.

## Introduction

### Historical development of the disease ontology in dermatology

In contrast to most other disorders, skin diseases can be observed by the eye. This is most likely why initial attempts to classify skin diseases were already made in the early days of the era of descriptional science, defined by the approach to group and classify biological species by their morphology. The most famous scientist to shape the era of descriptional science is Carl von Linné who proposed classifications of plant and animal biology in the 18th century. It was at the same time when Joseph Plenck (Holubar and Frankl, 1984) in 1776 created the first elementary ontology of skin diseases. This ontology mixed efflorescences such as macule, papule, or vesicle with pseudodescriptions such as growth and injuries. It was taken up and modified by several godfathers of dermatology such as Robert Willan (Tilles and Wallach, 1999; Willan, 1808) before it finally formed the basis for the first famous atlas of skin diseases authored by Ferdinand von Hebra in 1856 (Finnerud, 1952). In his “Atlas der Hautkrankheiten,” Hebra distinguished 12 classes of skin diseases such as exsudations, atrophias, benign or malignant growths, and ulcers. His classification up to date is the basis for many standard textbooks in dermatology (Jackson, 1977). Today, these textbooks contain up to 3000 different diagnoses, and all of them are made subjectively by adding different diagnostic clues. These clues include the clinical description of the lesion(s); distribution and development over time; trigger factors; personal and family history of the patient; and eventually, laboratory work up such as the histological architecture of skin lesions, growth of pathogenic microorganisms, or detection of pathogenic antibodies in skin or circulation.

In some of the most common skin diseases such as atopic dermatitis (AD), diagnostic clues are defined by different associations or experts such as the United Kingdom working party, Hanifin and Rajka, or the American Association of Dermatology (Hanifin and Rajka, 1980; Wollenberg et al, 2022). For many other skin diseases, clear definitions do not currently exist, but even if a combination of diagnostic clues is defined that comprise a diagnosis, there is a tremendous heterogeneity of pathological skin conditions that actually fulfill the minimum requirements of these definitions. Furthermore, many patients show overlapping diagnostic clues. Even the differential diagnosis of 2 of the most common inflammatory skin diseases, namely AD and psoriasis, can be very difficult in certain localizations such as palms or scalp (Garzorz-Stark and Eyerich, 2019) or show mixed phenotypes, a situation that has been called “eczematized psoriasis” (Lauffer and Eyerich, 2023), “psoriasiform dermatitis,” or “eczema in psoriatico” (Kolesnik et al, 2018). Thus, the traditional disease classification in dermatology led to the description of several hundreds of diagnoses that are often ill defined and overlapping. It was made, however, in a time when we did not have profound knowledge of the immune system or basic biological events, and it still helps modern dermatologists to make therapeutic decisions. However, it is without doubt that the traditional disease ontology needs revision.

### Shifting from traditional toward stratified medicine in inflammatory skin diseases

Such a revision is seen in dermato-oncology, where the expanding knowledge on tumor-driving mutations and immune evasion strategies not only led to the development of targeted therapies and checkpoint inhibitors but also led to a revised classification of tumor entities based on molecular events, for example, in the field of melanoma (Elder et al, 2020), keratinocyte carcinomas (Conforti et al, 2019), or lymphoproliferative disorders (Kempf et al, 2024). It is also seen to some extent in the field of genodermatoses, where ichthyoses (Oji et al, 2010) or epidermolysis bullosa (Has et al, 2020) was reclassified according to the mutational cause in parallel to gene therapy developments.

So far, the revision of our disease ontology in the field of inflammatory skin diseases, however, is incomplete. That does not mean that technical developments did not lead to a better understanding of major pathogenic processes. In fact, major advances have been made in understanding the interaction of migrating immune cells and resident stromal and epithelial cells in inflammation. Today, the so-called immune response patterns can be defined. Although this concept of stratified medicine is simplifying and needs to be refined once more granular datasets are available, it allows the following grouping of the vast majority of polygenic inflammatory skin diseases (Eyerich and Eyerich, 2018; Schäbitz et al, 2022; Seiringer et al, 2022):

1. Pattern I, the cytotoxic pattern, is caused by type I immune cells such as cytotoxic CD8+ T cells, NK cells, or T helper 1 cells. These cells share the transcription factor T-bet and the secretion of the cytokine IFN-γ that induces Jak/signal transducer and activator of transcription–dependent cellular toxicity in target cells such as keratinocytes or melanocytes (Shao et al, 2019). Examples of type I diseases are lichen planus (Lauffer et al, 2018), cutaneous lupus, adverse drug reactions (Nordmann et al, 2024), graft-versus-host-disease, and alopecia areata and vitiligo.
2. Pattern II, caused by type II immune cells expressing GATA-3 and secreting IL-4, IL-5, IL-9, IL-13, IL-31, and other cytokines, is divided into cellular (2a) and humoral (2b) patterns. Pattern 2a is driven by impaired epidermal barrier due to altered keratinocyte differentiation, inhibited innate immune responses, and neuroimmunological changes leading to pruritus, and it occurs in AD (Ständer, 2021), nummular eczema (Böhner et al, 2023), contact dermatitis, and beyond; pattern 2b is caused by pathogenic (auto)antibodies and causes autoimmune blistering diseases such as pemphigus or bullous pemphigoid.
3. Pattern III is induced by RORc+ immune cells such as T helper 17 cells or innate lymphoid cells type 3 that secrete IL-17A, IL-17F, IL-21, or IL-22 and cause a wound-healing–like reaction in tissue, including enhanced metabolic and proliferative activity and an activated innate immune response and neutrophil granulocyte migration. It is observed in psoriasis and sister diseases but also in neutrophilic dermatoses or acne.
4. Pattern IV is driven by regulatory T cells that are characterized by the transcription factor Foxp3 and secretion of cytokines such as TGF-β or IL-10. TGF-β is crucial to diseases based on tissue remodeling and fibrosis, for example, scleroderma or eosinophilic fasciitis; pattern 4a occurs primarily or as a consequence of a preceding inflammation (Seiringer et al, 2022). Least evidence exists for pattern 4b, where a disbalance of pro and anti-inflammatory cytokines may cause the development of granulomas, observed, for instance, in granuloma annulare or sarcoidosis.

This classification based on immune response patterns is both clinically meaningful and simplifying. It follows the concept of stratified medicine that groups patients with a higher likelihood to respond to specific therapies (Litman, 2019), but it cannot give detailed information about an individual patient. Nevertheless, this concept allows to expand the disease ontology beyond the classical diagnostic clues, because molecular classifiers can be established. On the basis of gene or protein expression in lesional skin, these classifiers give objective information about the underlying immune response patterns. An early classifier distinguishing type 2 from type 3 immune patterns consists of the 2 genes *NOS2* and *CCL27* (Fischer et al, 2023; Garzorz-Stark et al, 2016; Quaranta et al, 2014). It is already used as an additional diagnostic tool in daily clinical routine at some expert centers. A recent study proposes a classifier that spans all known immune-response patterns and adds neutrophil, eosinophil, and type 1 IFN modules on the basis of 600 immune-related genes in lesional skin (Seremet et al, 2024). Using both classifiers improves the therapeutic outcome compared with using the traditional diagnostic clue criteria (Seremet et al, 2024).

### Shortcomings of the current classification

The proposed classification of inflammatory skin diseases according to immune response patterns or immune modules provides a rationale to predict which therapy is the most promising in an individual patient. Pattern I diseases may be treated with Jak inhibitors; pattern II diseases may be treated most specifically with IL-4Ra or IL-13 antibodies; and pattern III diseases may be treated with TNF-α, IL-17, or IL-23 inhibitors. Pattern IV diseases are generally more difficult to treat, but TNF-α inhibitors may be used against granulomatous diseases, and the anti-CD20 antibody rituximab has shown good effects in systemic fibrosing diseases such as sclerosis.

Nevertheless, fundamental questions of precision medicine remain open despite all efforts made so far, for example, what is the likelihood to respond to a given therapy? Even the most efficient biologic therapies leave a substantial number of nonresponders. In the field of psoriasis, nonresponders are at least 15% of the entire patient population (Armstrong et al, 2022). In the field of AD, at least 25% are nonresponders (Wollenberg et al, 2018). In other inflammatory skin diseases, this population is typically even larger. Furthermore, a subpopulation of patients maintains disease control upon extended drug intervals (Eyerich et al, 2024a; Worm et al, 2020) or even after drug withdrawal far beyond pharmacological effects (Gordon et al, 2019; Krueger et al, 2024). It is impossible to predict at an individual level which patients may achieve such a disease modification (Eyerich et al, 2024b). Finally, some patients show a secondary loss of efficacy under treatment. Despite the fact that there may be a weak correlation with development of neutralizing antidrug antibodies in the case of TNF inhibitors (Anjie et al, 2024), there is no model to predict which patient will suffer from such a weaning efficacy.

What is the risk to develop a drug-related adverse event? Even though modern therapies of inflammatory skin diseases overall have a good safety profile, drug-related adverse events have been recorded (Trefzer et al, 2024), for example, conjunctivitis in up to 25% of all patients with AD treated with biologics such as dupilumab (Halling et al, 2021; Thyssen et al, 2019), up to 15% of all patients with AD developing acne-like syndromes when treated with Jak inhibitors such as upadacitinib (Blauvelt et al, 2021), or up to 10% of all patients with psoriasis developing mucocutaneous candida infections when treated with IL-17 inhibitors such as bimekizumab (Strober et al, 2023). Furthermore, 2–5% of patients with psoriasis treated with biologics may develop AD-like lesions, and vice versa, patients with AD may develop psoriasiform plaques upon treatment (Al-Janabi et al, 2020; Trefzer et al, 2024). It is currently not possible to precisely predict which patient is likely to develop any kind of adverse event.

What is the risk to develop comorbidity? Inflammatory skin diseases are frequently associated with comorbidities such as cardiovascular abnormalities, metabolic syndrome, or psychosomatic disorders, and they may precede arthritis in the case of psoriasis or allergic asthma and rhinoconjunctivitis in the case of AD (Brunner et al, 2017). Recent evidence suggests that the development of comorbidities may be prevented using efficient therapies as early as possible (Gisondi et al, 2022; Lin et al, 2024), but it is currently impossible to predict which patient would develop a comorbidity or benefit from an early treatment.

What is the natural clinical course of an inflammatory skin disease? The prevalence of AD is much higher in children than in adults; thus, there is the tendency to self-limited inflammation in a subpopulation of children with AD (Langan et al, 2020). There are approaches to predict in which patients AD may persist on the basis of historical and serum diagnostic clues (Lauffer et al, 2021), changes in cutaneous microbiome (Fonfara et al, 2024), or genetic phenotype (Margolis et al, 2012), but no validated predictive system exists as of now. Likewise, the evolution from mild to moderate or severe disease may happen in numerous inflammatory skin diseases, including psoriasis, but also this evolution of disease is unpredictable at an individual´s patient level.

## Materials and Methods

### Hypothesis and study objectives

**Figure 1:**
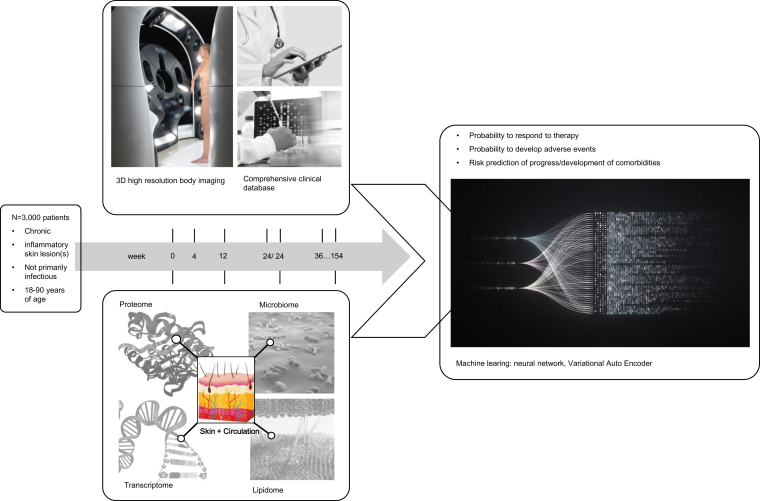
**The approach of the TD initiative: integrating clinical phenotyping and multiomics data over time in a large population of patients spanning the whole spectrum of inflammatory and autoimmune skin diseases.** TD, translational dermatology.

The central hypothesis of the translational dermatology (TD) initiative is that precision medicine in inflammatory skin diseases is not achieved owing to shortcomings in our disease ontology. The current disease nomenclature is highly variable, and diagnostic work up ranges from clinically subjective description to molecular and genetic testing. Furthermore, neither disease heterogeneity nor dynamics are sufficiently addressed. It is further hypothesized that these shortcomings can be overcome by integrating deep clinical phenotyping and molecular analyses of inflammatory skin diseases over time using machine-learning algorithms independent of the traditional disease diagnosis (Figure 1). Because the approach is centered around a comprehensive dataset at a given time point of an individual patient and disregard the static historical disease categorization, dynamics of the inflammatory pathogenesis, including development of adverse events such as paradoxical phenotypes to biological therapies, are addressed (Trefzer et al, 2024).

The objective of the TD initiative is to create the basis for a new disease ontology of inflammatory skin diseases on the basis of objective molecular events that enables the clinician to answer clinically relevant questions, such as individual probability of treatment success, risk to develop an adverse event, and clinical need for treatment defined by risk to develop comorbidities versus tendency of self-limited natural clinical course.

### Integrating phenotyping and molecular data: the TD concept study design

The TD initiative is a prospective, noninterventional, multicenter clinical registry comprising full-body imaging and molecular analysis from circulation, nonlesional, and lesional skin over time (Table 1). Patients will be characterized at baseline, at weeks 4 and 12, and then at each 6 months in an unlimited design. Furthermore, patients will be seen upon a substantial change of the medical condition defined as need for change of treatment. At baseline, patients are required to meet the following inclusion criteria:

• Chronic disease, defined as >6 weeks of skin lesions;
• Inflammatory skin lesion(s); and
• Being aged 18–90 years.

**Table 1:** Flow Chart of the TD Initiative

Data	Visit 1 (wk 0)	Visit 2 (wk 4)	Visit 3 (wk 12)	Visit 4 (mo 6)	Consecutive Visit I^1^	Consecutive Visit II^2^	Additional visit^3^
Notes	x	x	x	x	x	x	x
Basic information							
Basic information	x				x		
Recent treatment	x	x	x	x	x	x	x
Patient assessment							
Scores general (IGA, BSA)	x	x	x	x	x	x	x
Disease-specific scores	x	x	x	x	x	x	x
Imaging							
Vectra 360	x	x	x	x	x	x	x
Consent form	x		x				x
Survey							
Basic information patient	x				x		
DLQI, PGA, Peak Pruritus NRS, global satisfaction, happiness	x	x	x	x	x	x	x
NEO-FFI, CD-RISC-10, HADS	x				x		
POEM (or other disease-specific survey)	x	x	x	x	x	x	x
Diagnosis (ICD)	x	x	x	x	x	x	x
Biobank							
Biopsy (cryo/RNA/FFPE) lesional	x		x				x
Biopsy (cryo/RNA/FFPE) nonlesional	x						
Blood storage EDTA (5 ml)	x		x		x		x
Blood storage serum (2 × 8 ml)	x	x	x	x	x		x
Lipidomics lesional/nonlesional	x		x		x		x
Proteomics lesional/nonlesional	x		x		x		x
Microbiome lesional/nonlesional	x		x		x		x
PBMCs (EDTA, whole blood)	x		x				x
Laboratory analyses							
Complete blood count	x	x	x	x	x		x
CRP	x	x	x	x	x		x
Liver, kidney	x				x		
Hba1c	x				x		
Lipids	x				x		
IgE	x				x		

Abbreviations: BSA, body surface area; CD-RISC-10, Connor–Davidson Resilience Scale 10; DLQI, Dermatology Life Quality Index; FFPE, formalin fixed, paraffin embedded; HADS, Hospital Anxiety and Depression Scale; ICD, International Classification of Diseases; NEO-FFI, NEO Five-Factor Inventory; NRS, Numerical Rating Scale; PGA, Physician Global Assessment; POEM, Patient-Oriented Eczema Measure; TD, translational dermatology.

1 Every 12 months; first time 6 months after visit 4.

2 Every 12 months; first time 6 months after consecutive visit I.

3 If change of clinical condition/medication change; same as visit 3.

The following are the exclusion criteria:

• Primarily infectious disease;
• Inability or unwillingness to undergo repeated venipuncture (eg, because of poor tolerability or lack of access to veins);
• Inability or unwillingness to undergo repeated punch biopsies;
• Any immune-active systemic treatment within 12 weeks or immune-active topical treatment within 2 weeks before inclusion; and
• Inability or unwillingness to understand and sign the written informed consent and/or the questionnaires.

Patients will answer a variety of questionnaires at different time points to assess to what degree the QOL is affected and how resilient and adherent a patient is to therapy (Table 1). Questionnaires include the Dermatology Life Quality Index, Hospital Anxiety and Depression Scale, Connor–Davids Resilience Scale, and Visual Analog Scales of peak pruritus, pain, and happiness. Questionnaires are complemented by physician-assessed scores such as Physician Global Assessment and body surface area as well as disease-specific scores such as PASI, Eczema Area and Severity Index, Cutaneous Lupus Area and Severity index, SCORing Atopic Dermatitis, Weekly Urticaria Activity Score, Bullous Pemphigoid Disease Area Index, Pemphigus Disease Area Index, and International Hidradenitis Suppurativa Severity Score. At baseline, a personality questionnaire, the NEO Five-Factor Inventory, is also answered by the patients. All questionnaires are completed digitally and added to the system live.

In addition to the questionnaires, a detailed medical history regarding previous treatments, comorbidities, and side effects of treatment is taken. Furthermore, image data are collected and stored from each patient at each visit. This is implemented using a high-resolution full-body imaging tool, the Canfield Vectra WB360 Imaging System.

Besides clinical data such as height, weight, and blood pressure, comorbidities will be asked for. Comorbidities of special interest include cardiovascular and metabolic disease, inflammatory rheumatology diseases or diseases of the gastrointestinal system, atopic comorbidities such as allergic asthma, and psychological comorbidities.

Finally, 6-mm skin punch biopsies are obtained from the patients (at visit 1 both from nonlesional and lesional skin, at visit 3 from [previously] lesional skin). Skin biopsies are cut into 3 pieces: (i) one piece will be formalin fixed and paraffin embedded for histological analysis (H&E stainings and immunohistochemical stainings at the discretion of the dermatopathologist), (ii) another piece will be snap frozen in liquid nitrogen (potential analyses of spatial transcriptomics, metabolomics, and proteomics at later steps), and (iii) the last piece is stored in RNA-stabilizing solution for the purpose of bulk RNA sequencing. Smear tests and tape strips used for microbiome shot-gun sequencing and lipidome or proteome (high-performance liquid chromatography) analyses, respectively, are also obtained from the same or closely adjacent sites of biopsy (lesional and nonlesional). Finally, approximately 40-ml venous blood will be taken from the patients at prespecified visits. From blood, PBMCs as well as serum and EDTA blood are stored in a biobank. Serum is used for proteome analysis. Routine blood tests are performed to monitor hematological, kidney, and liver parameters as well as CRP and other analyses.

In case of changes of the clinical condition (eg, deterioration of symptoms, change of medication) of a participant, additional visit(s) will be conducted, and clinical assessment, questionnaires, and whole-body photography will be repeated. In addition, additional biological samples of lesional skin will be taken, including skin biopsy, microbiome swabs, proteome and lipidome tape strips, blood, and serum. Additional skin biopsies may however only be repeated a further 2 times per patient in addition to the sampling at visits 1 and 3.

All data are stored and collected using a tailored database optimized for machine learning. The TD database is an inhouse data-collection platform developed as a part of this project to facilitate bias-free collection and analysis of diverse large-scale data from clinical trials. This database allows customized questionnaires and visits and adds images and molecular data to a given visit of a given patient. The database also has a biobank functionality and holds all data-protection standard measurements, including GDPR (General Data Protection Regulation) (further information can be found in Supplementary Material).

### Machine-learning and biocomputational approach

Data-driven endotypes are defined by leveraging large-scale datasets and computational methods to identify clinically meaningful patient clusters. These clusters or endotypes are defined on the basis of underlying biological mechanisms and clinical outcomes rather than predefined clinical criteria (Angelini et al, 2022). The approach uses unsupervised learning, clustering, and statistical models to identify and uncover patterns and biomarkers that define and characterize distinct endotypes.

In this study, diverse types of data from patients are collected, including questionnaires, physician-based scores, images, and molecular data. To utilize different computational techniques to derive distinct endotypes, combination of different information cues into a comprehensive data source can be challenging (Williams-DeVane et al, 2013). The approach taken in this study will rely on using intermediate layers of an end-to-end neural network as the descriptor for the samples aggregating different cues of information. This intermediate layer can be interpreted as a mapping of the original data into a lower dimensional space, which can either be part of a Variational Auto Encoder trained in an unsupervised manner (Ilnicka and Schneider, 2023) or be part of a neural network trained to predict various clinical attributes.

### Status and perspectives

The TD initiative creates a high-quality dataset of subjective and objective clinical assessment, image data, and multiomics data from circulation, nonlesional, and lesional skin over time from patients covering the entire spectrum of inflammatory skin diseases. It is run at identical materials and protocols at the Department of Dermatology and Venerology of the Medical Center at the University of Freiburg (Freiburg, Germany) and the Division of Dermatology and Venerology at the University Hospital Karolinska (Stockholm, Sweden). Because the thorough analysis of patients is cost intense in the dimension of up to 10,000 Euro per patient, the TD initiative relies on public funding as well as support from industry partners. Currently, several hundred patients with diverse inflammatory skin diseases have been included, with the aim to recruit at least 3000 patients in total. Given the fact that ethnicity may influence the pathogenesis of inflammatory skin diseases, it is desirable to expand the TD initiative beyond Europe by including centers in the Americas, Asia, Australia, and Africa.

## Ethics Statement

The study was approved by the local ethics committees of the Medical Center of the University of Freiburg (22-1034) and the University Hospital Karolinska (2020-04125). All participants provided written and informed consent prior to inclusion in the study.

## Data Availability Statement

All data of this article are freely available upon request to the corresponding author (kilian.eyerich@uniklinik-freiburg.de).

## ORCID

Kilian Eyerich: http://orcid.org/0000-0003-0094-2674

## Conflict of Interest

The authors state no conflict of interest.
