# Supplementary material for: The Translational Dermatology Initiative: Aiming at a New Disease Classification of Inflammatory Skin Diseases
Source: JID Innov. 2025 May 13;5(5):100381. doi: 10.1016/j.xjidi.2025.100381 (PMC12173067; doi:10.1016/j.xjidi.2025.100381)

Translational Dermatology Database

The holistic software solution to enhance research
for personalized medicine

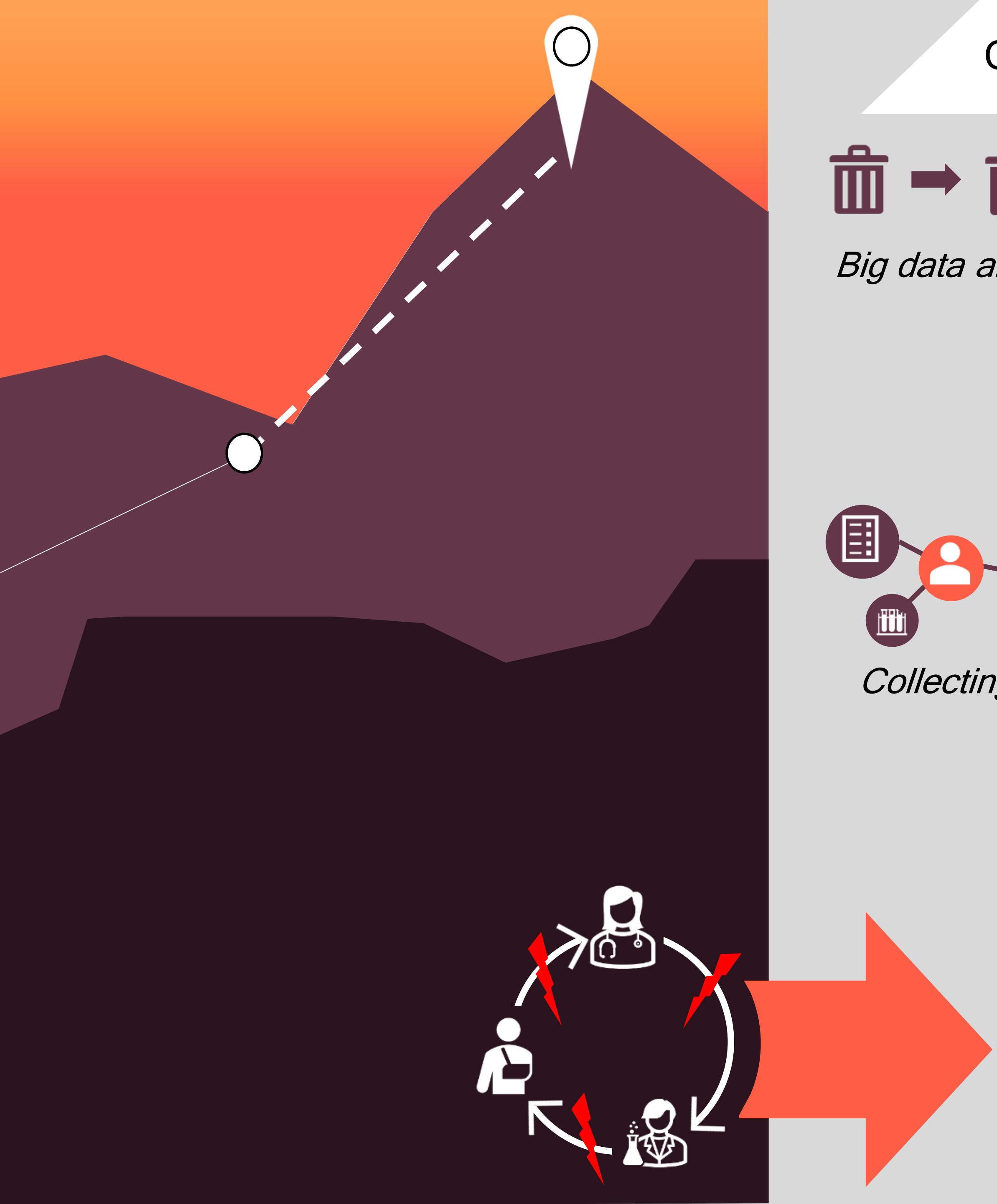

Current obstacles standing against precision medicine

Data collection

Big data algorithms are only as good as the data they handle!

- Observer bias
- Different data collected over time
- Incomplete data
- Time and money consuming collection of data (various assessment tools)

Data availability and handling

Collecting data for scientific purposes is still a Sisyphean task!

- Unstructured storage of complex data
- Data often only available paper-based
- Lost data
- Time and money consuming search of data (various unharmonized data storages)

Data analysis:

- Lack of high quality data sets
- Overfitted and unrepresentative *in silico* models
- Failed validation *in vitro*
- Prolonged or even missed introduction of individual concepts in clinical practice

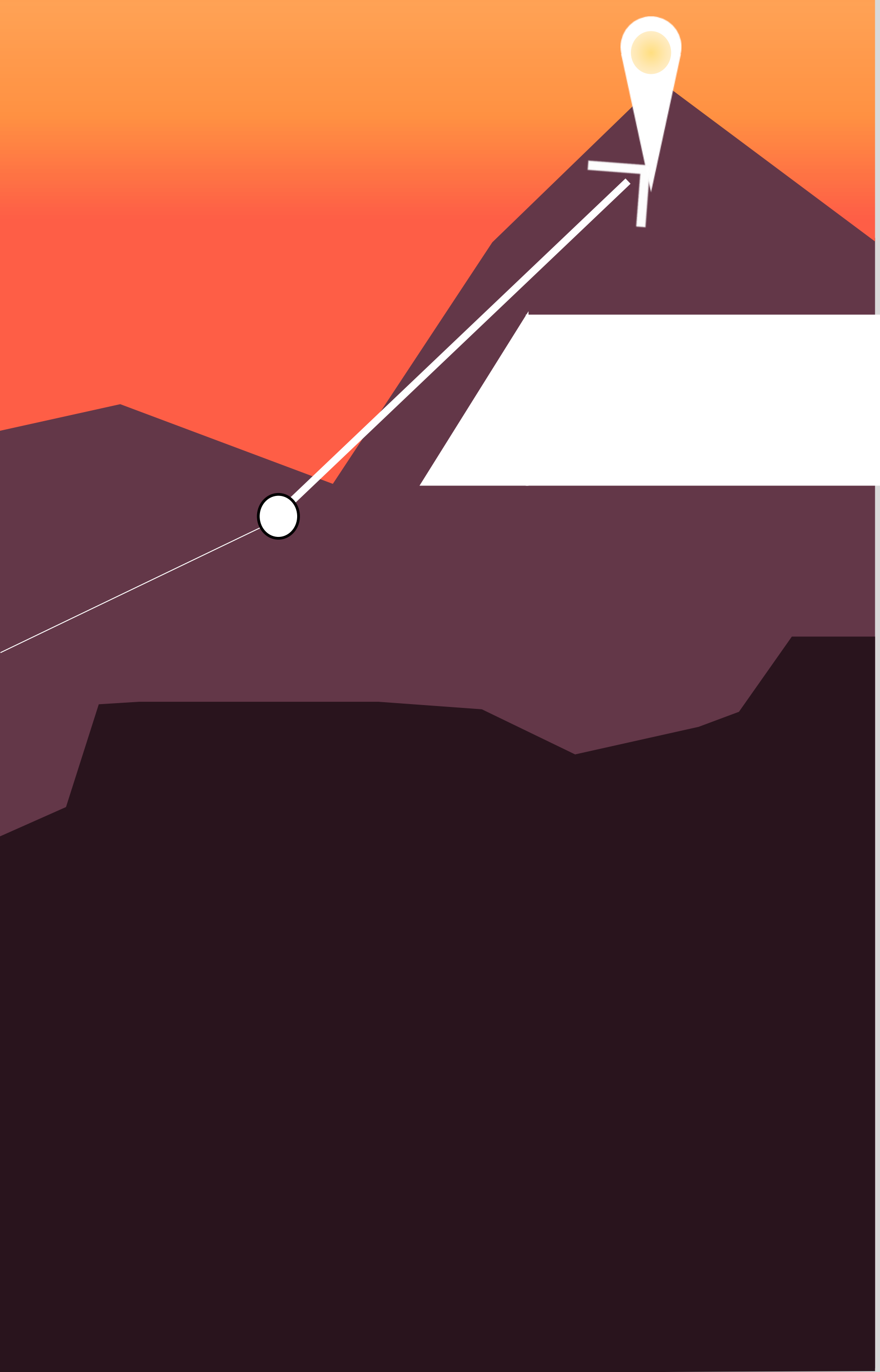

TD database - advantages

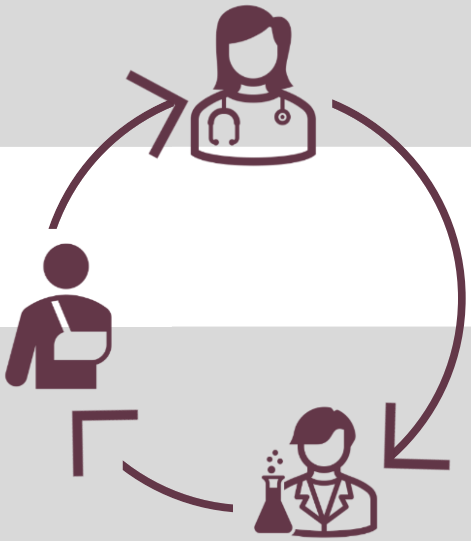

Designed for clinician scientists by clinician scientists to enable efficient research for personalized medicine

- **All-in-one solution**: Seamless and comprehensive collection, handling and analysis of highly dimensional complex data (360° Canfield images, OMICs data, questionnaires…)
- **Userfriendly Operation**: Intuitive interface, time-efficient use in stressful environments
- **Customizable**: 100%-customizable by minimally trained user without external support
- **High quality data**: Standardized data collection for individual entity‘s research and collaborative projects
- **Affordable**: All modules included, highly competitive pricing based on data volume
- **Data privacy**: Customer owns data and data is stored on customer‘s own server

TD database - How does it work?

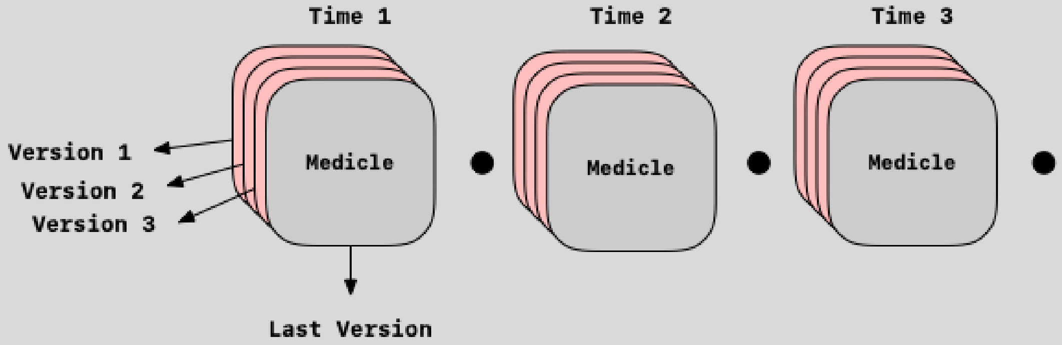

Medicle :
- Smallest block of data
- Stores a piece of medical data
- Changes are versioned
- Tracking the same data over time

Examples :
- General/Basic Information
- Image
- Blood Measurement
- Biological Sample
- Survey Score
- Answer to a specific question from a survey
- Etc.

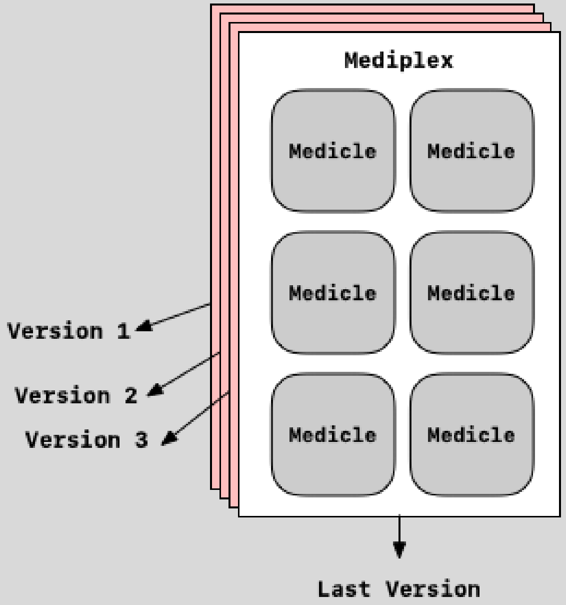

Mediplex :
- Collection of **dependent** Medicles
- All Medicles are versioned together
- Data is collected over different visits
- Individual Medicles can be tracked

Examples :
- Surveys/Questionnaires
- Patient Information
- Etc.

TD database - How does it work?

Data collection

- Standard Medicles and Medules are already available
- Individual Medicles and Medules can be easily created
- Integrated automized software checks enable medule consistency and further downstream analysis
- Patient can be directly involved in digital data collection by tablet portal (data only touched once!)

Data handling and storage

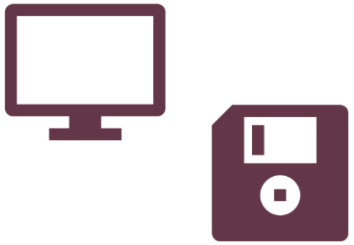

- Frontend software, backend software and data base run decentralized on customer‘s environment
- GDPR, GCP standards fulfilled (password protected, double pseudonymized and versioned data)
- Full tracability of data by blockchain
- Integrated biobank function and lab book function

Data search and analysis

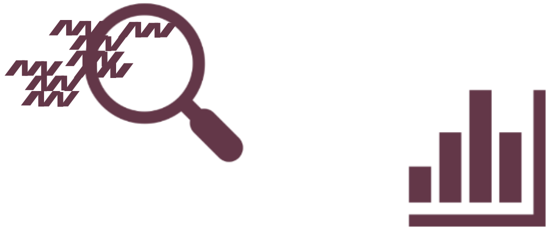

- Data search along medules for clinical studies and wet lab research
- Standard data analysis with statistical programmes (SPSS, graphpad functions)
- Proven machine learning tools for highly dimensional data and complex analyses

Data collection

Choose/design the information that should be collected

Design how the information is going be collected

Collect and store the information

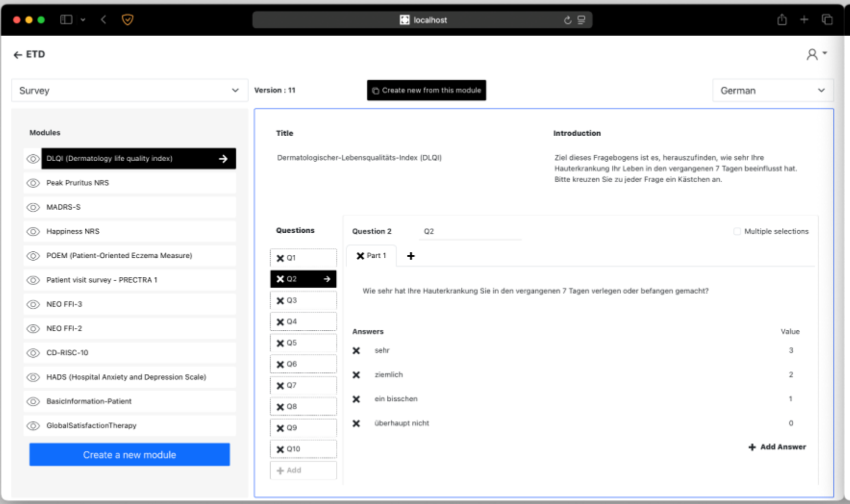

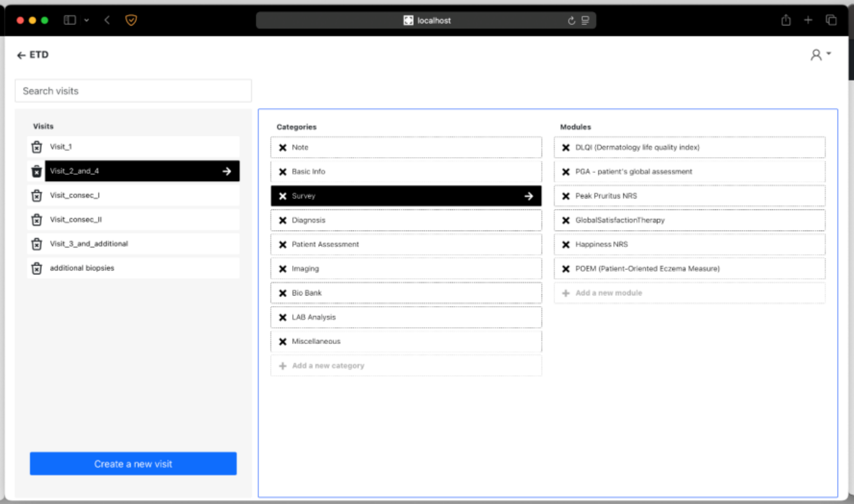

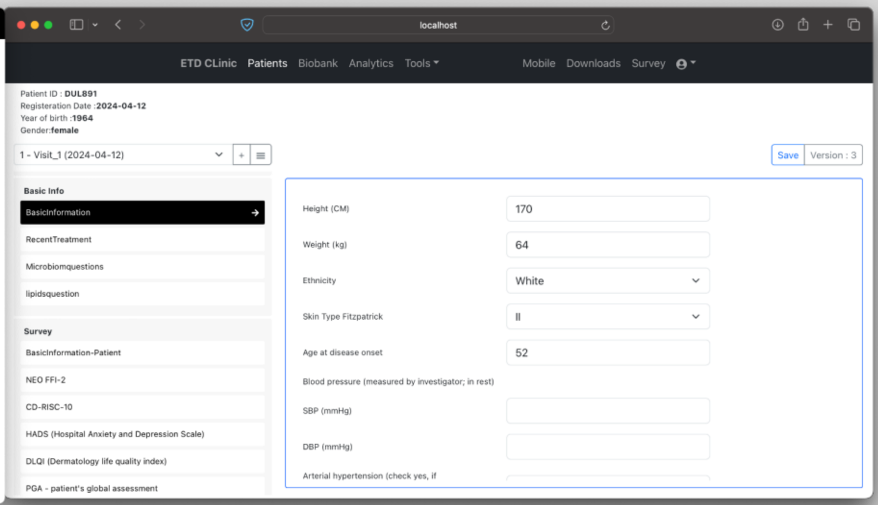

Module Builder

Visit Designer

Data Collection

Data collection

Collect, register and track bio-samples

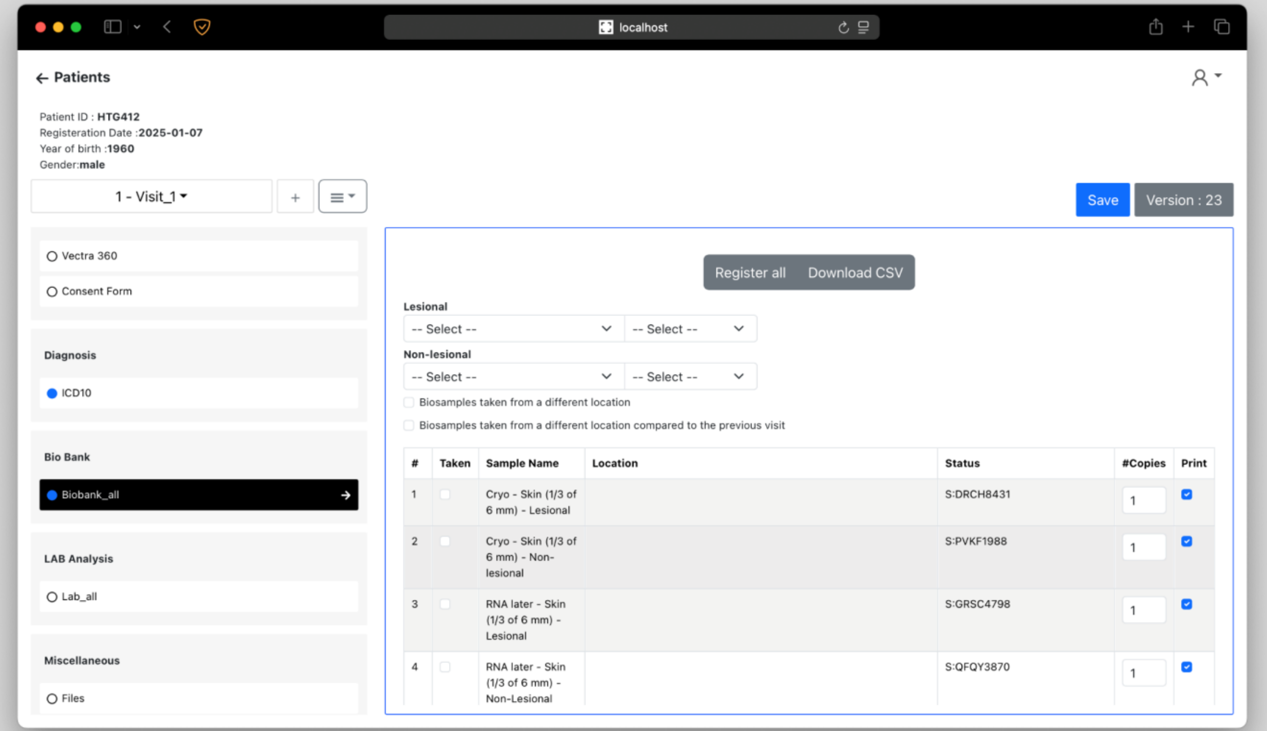

Biobank Entry
(Visits)

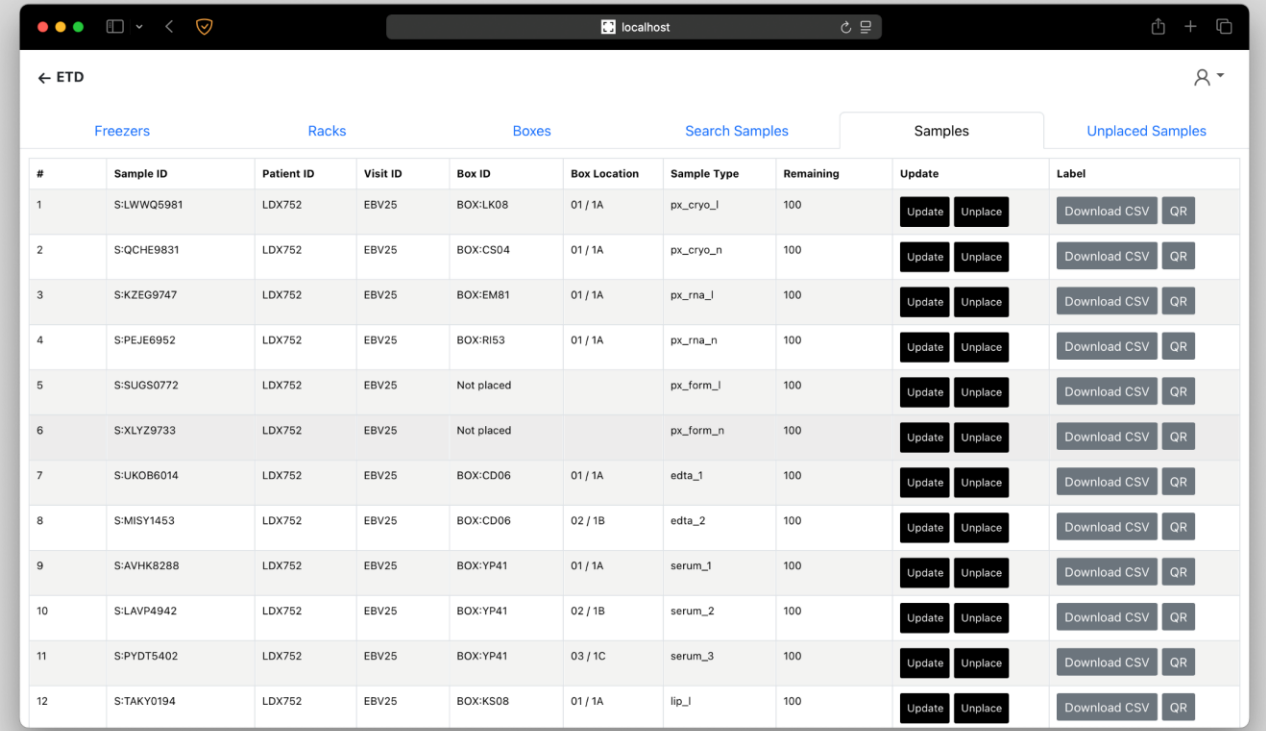

#	Sample ID	Patient ID	Visit ID	Box ID	Box Location	Sample Type	Remaining	Update	Label
1	S:LWWQ5981	LDX752	EBV25	BOX:LK08	01 / 1A	px_cryo_l	100	Update Unplace	Download CSV QR
2	S:QCHE9831	LDX752	EBV25	BOX:CS04	01 / 1A	px_cryo_n	100	Update Unplace	Download CSV QR
3	S:KZEG9747	LDX752	EBV25	BOX:EM81	01 / 1A	px_rna_l	100	Update Unplace	Download CSV QR
4	S:PEJE6952	LDX752	EBV25	BOX:RI53	01 / 1A	px_rna_n	100	Update Unplace	Download CSV QR
5	S:SUGS0772	LDX752	EBV25	Not placed		px_form_l	100	Update Unplace	Download CSV QR
6	S:XLYZ9733	LDX752	EBV25	Not placed		px_form_n	100	Update Unplace	Download CSV QR
7	S:UKOB6014	LDX752	EBV25	BOX:CD06	01 / 1A	edta_1	100	Update Unplace	Download CSV QR
8	S:MISY1453	LDX752	EBV25	BOX:CD06	02 / 1B	edta_2	100	Update Unplace	Download CSV QR
9	S:AVHK8288	LDX752	EBV25	BOX:YP41	01 / 1A	serum_1	100	Update Unplace	Download CSV QR
10	S:LAVP4942	LDX752	EBV25	BOX:YP41	02 / 1B	serum_2	100	Update Unplace	Download CSV QR
11	S:PYDT5402	LDX752	EBV25	BOX:YP41	03 / 1C	serum_3	100	Update Unplace	Download CSV QR
12	S:TAKY0194	LDX752	EBV25	BOX:KS08	01 / 1A	lip_l	100	Update Unplace	Download CSV QR

Biobank
Sample Collection and Tracking

Data export

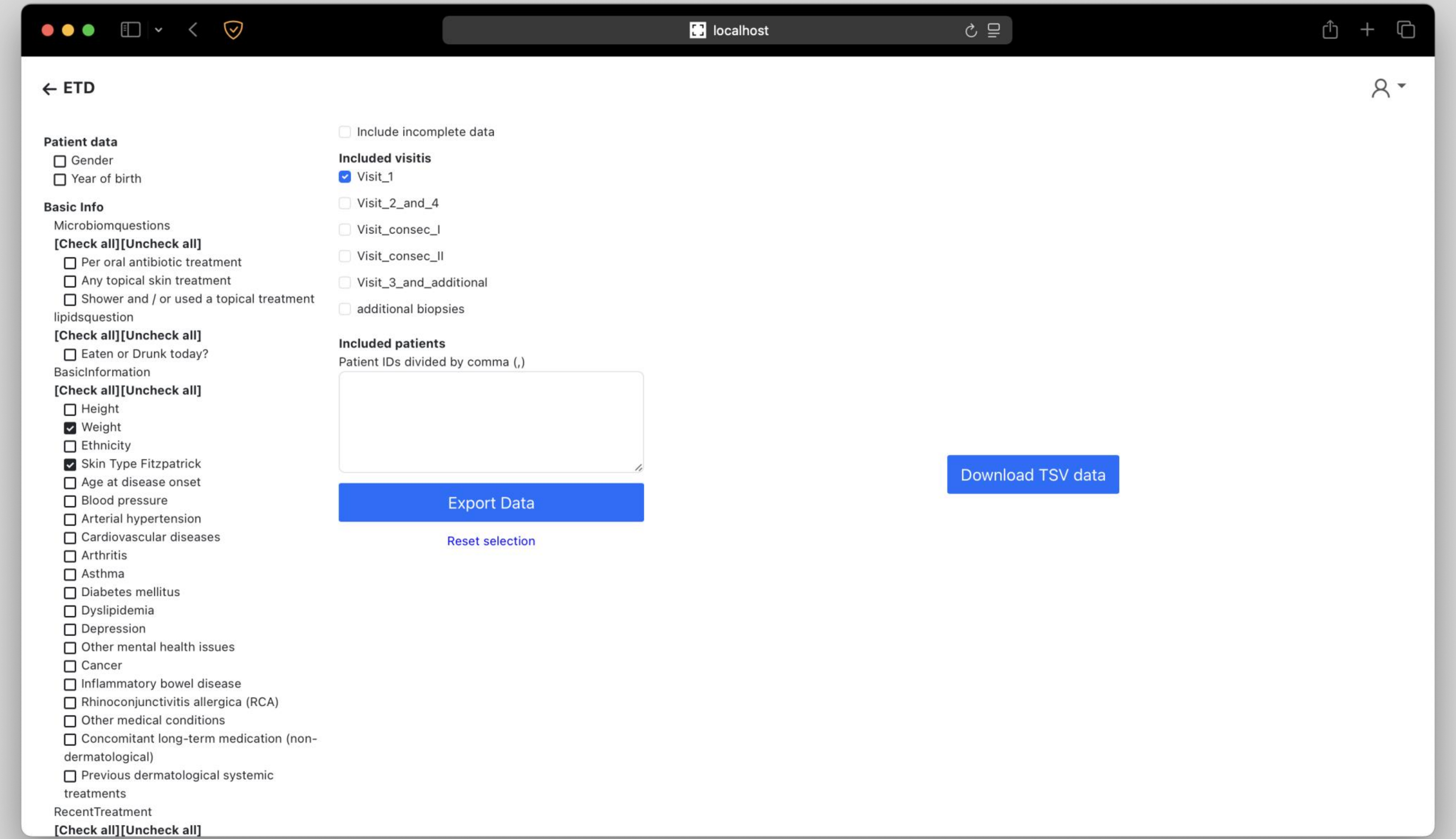

Easily download part of the dataset for further analysis

Data analysis and visualization

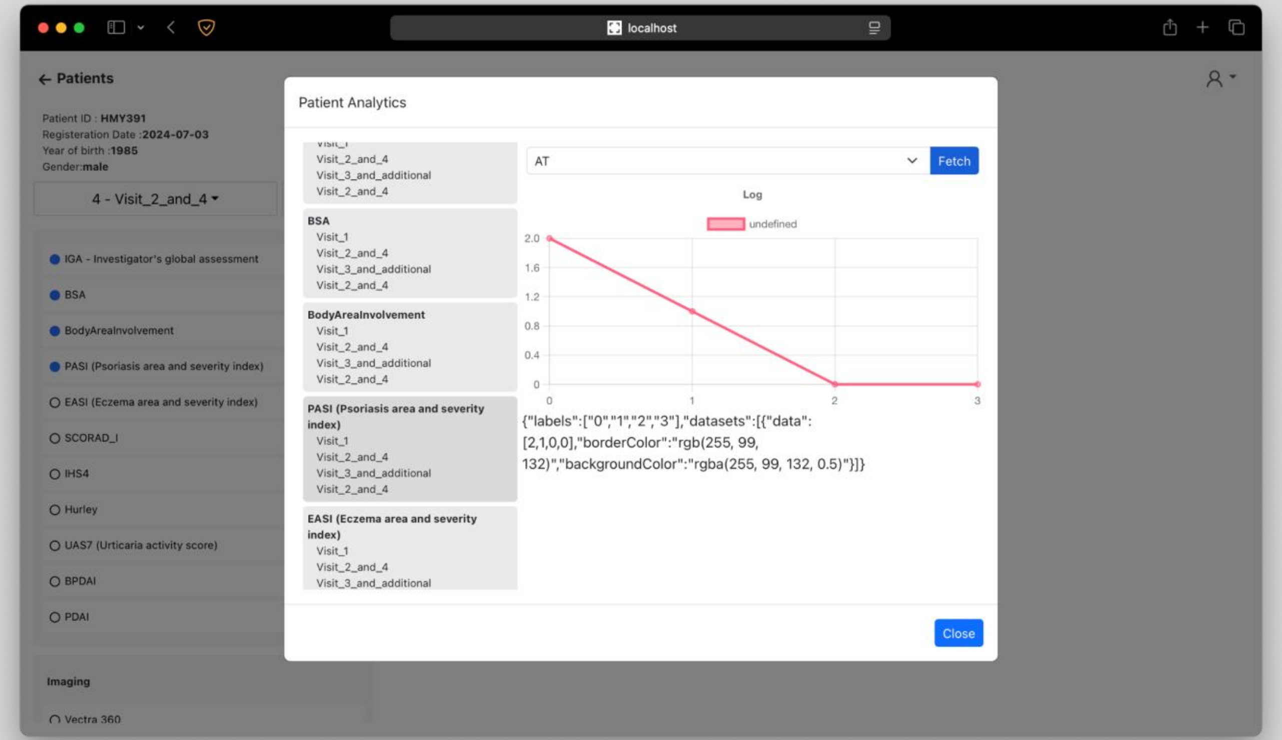

Use built-in tools to track and visualize the data for each patient

Use built-in data analysis toolbox to apply Machine Learning tools the data (Coming soon)

Data handling and storage

2.1) Large data storage

- Storing and backing-up data on customer‘s storage solution, e.g. S3 storage; all data in customer‘s hands and not on provider‘s clouds
- Data responsibility and rights in customer‘s hands
- Blockchain technologies to ensure data consistency and tracabilty

2.2) **Small data storage** (small data and references to big data in large data storage)

- SQL data base

2.3) **Solution packaging**

- frontend/backend/database as docker containers for easy software implementation at customer‘s place
- Software is provided in compiled form to customers; source code remains fully in company‘s hands

Data handling and storage

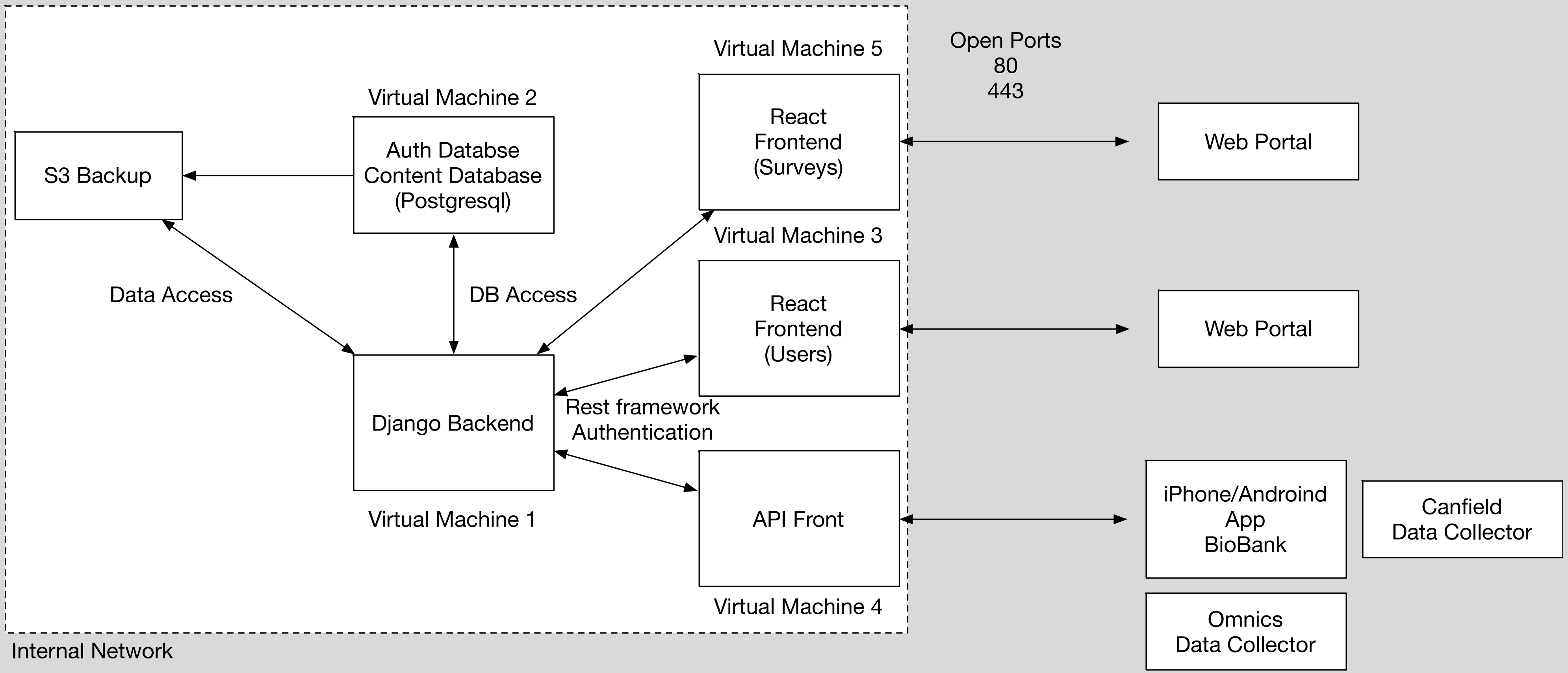

Supplement: Supplementary Material [file mmc1.pdf]
